# Comparative Effects of Deferiprone and Salinomycin on Lead-Induced Disturbance in the Homeostasis of Intrarenal Essential Elements in Mice

**DOI:** 10.3390/ijms23084368

**Published:** 2022-04-15

**Authors:** Yordanka Gluhcheva, Irena Pashkunova-Martic, Martin Schaier, Ivelin Vladov, Silviya Stoykova, Emilia Petrova, Ekaterina Pavlova, Peter Dorkov, Thomas H. Helbich, Bernhard Keppler, Juliana Ivanova

**Affiliations:** 1 Institute of Experimental Morphology, Pathology and Anthropology with Museum, Bulgarian Academy of Sciences, Acad. Georgi Bonchev Street, Bl. 25, 1113 Sofia, Bulgaria; ygluhcheva@hotmail.com (Y.G.); iepparazit@yahoo.com (I.V.); emiliapetrova@abv.bg (E.P.); e_bankova@yahoo.com (E.P.); 2 Department of Biomedical Imaging and Image-Guided Therapy, Division of Molecular and Structural Preclinical Imaging, Medical University of Vienna and General Hospital of Vienna, 18–20 Waehringer Guertel, 1090 Vienna, Austria; irena.pashkunova-martic@meduniwien.ac.at (I.P.-M.); thomas.helbich@meduniwien.ac.at (T.H.H.); 3 Institute of Analytical Chemistry, University of Vienna, 38 Waehringer Strasse, 1090 Vienna, Austria; m.schaier@web.de; 4 Faculty of Chemistry and Pharmacy, Sofia University “St. Kliment Ohridski”, 1 J. Bourchier Blvd., 1164 Sofia, Bulgaria; ahss@chem.uni-sofia.bg; 5 Chemistry Department, Research and Development, BIOVET JSC, 39 Peter Rakov Street, 4550 Peshtera, Bulgaria; p_dorkov@abv.bg; 6 Institute of Inorganic Chemistry, University of Vienna, 42 Waehringer Strasse, 1090 Vienna, Austria; bernhard.keppler@univie.ac.at; 7 Faculty of Medicine, Sofia University “St. Kliment Ohridski”, 1 Kozjak Street, 1407 Sofia, Bulgaria

**Keywords:** deferiprone, salinomycin, lead, essential elements, kidneys

## Abstract

Lead (Pb) exposure induces severe nephrotoxic effects in humans and animals. Herein, we compare the effects of two chelating agents, salinomycin and deferiprone, on Pb-induced renal alterations in mice and in the homeostasis of essential elements. Adult male mice (*Institute of Cancer Research* (*ICR*)) were randomized into four groups: control (Ctrl)—untreated mice administered distilled water for 28 days; Pb-exposed group (Pb)—mice administered orally an average daily dose of 80 mg/kg body weight (BW) lead (II) nitrate (Pb(NO_3_)_2_) during the first two weeks of the experimental protocol followed by the administration of distilled water for another two weeks; salinomycin-treated (Pb + Sal) group—Pb-exposed mice, administered an average daily dose of 16 mg/kg BW salinomycin for two weeks; deferiprone-treated (Pb + Def) group—Pb-exposed mice, administered an average daily dose of 20 mg/kg BW deferiprone for 14 days. The exposure of mice to Pb induced significant accumulation of the toxic metal in the kidneys and elicited inflammation with leukocyte infiltrations near the glomerulus. Biochemical analysis of the sera revealed that Pb significantly altered the renal function markers. Pb-induced renal toxicity was accompanied by a significant decrease in the endogenous renal concentrations of phosphorous (P), calcium (Ca), copper (Cu) and selenium (Se). In contrast to deferiprone, salinomycin significantly improved renal morphology in Pb-treated mice and decreased the Pb content by 13.62% compared to the Pb-exposed group. There was also a mild decrease in the renal endogenous concentration of magnesium (Mg) and elevation of the renal concentration of iron (Fe) in the salinomycin-treated group compared to controls. Overall, the results demonstrated that salinomycin is a more effective chelating agent for the treatment of Pb-induced alterations in renal morphology compared to deferiprone.

## 1. Introduction

Chronic kidney disease (CKD) is considered one of the leading causes of death around the world [1]. Data revealed that a lead concentration in the blood of even below 5 microgram/dL could contribute to nephrotoxicity and increase the progression of CKD in diabetic and non-diabetic patients [2,3,4]. Studies on Pb-exposed animals have unequivocally indicated that Pb induces severe renal injury [5,6]. One of the mechanisms by which Pb can induce tissue injury is related to the alteration of the homeostasis of essential elements [7,8,9].

Therapy with chelating agents is a medical approach to the treatment of metal poisoning. The main drawback of this procedure is that the chelating agents and their metal complexes are nephro- and hepatotoxic [10]. Moreover, the administration of chelating agents alters the endogenous concentrations of essential metal ions [11].

Calcium disodium edetate (CaNa_2_EDTA) and meso 2-3-dimercaptosuccinic acid (DMSA) have been used as chelating agents for the treatment of Pb poisoning [12]. In vivo studies demonstrated that the polyether ionophorous antibiotic monensin increased the effectiveness of DMSA and its derivative as antidotes for the treatment of Pb toxicity [13,14]. Previously published studies compared the effects of monensin and the polyether ionophorous antibiotic salinomycin on Pb content in the organs of Pb-exposed mice [15,16] and revealed that salinomycin was more effective compared to monensin in mobilization of Pb from the brain [15]. To date, there are no data in the literature about the effect of salinomycin on Pb-induced renal toxicity.

Among the oxygen-containing ligands, deferiprone attracted the attention of researchers as a potential antidote for Pb intoxication [17,18]. Deferiprone is an oral chelating agent with low toxicity, approved by the U.S. Federal Drug Administration (FDA) for the treatment of thalassemia major. Studies on Pb-exposed rats have demonstrated that this chelating agent inhibited Pb-induced oxidative stress in the liver [17] and significantly decreased the toxic metal ion in some other organs [18]. To our knowledge, there are still no data about the effect of deferiprone on Pb-induced alterations in the renal histoarchitecture and the homeostasis of essential elements. 

Herein, we compare the effects of deferiprone and salinomycin on the Pb-induced disturbance in the homeostasis of essential elements in the kidneys of Pb-exposed mice. The influence of both chelating agents on the Pb-induced alterations of serum renal functional markers and renal histoarchitecture of Pb-exposed mice is also evaluated.

## 2. Results

### 2.1. Effects of Deferiprone and Tetraethylammonium Salt of Salinomycinic Acid (Salinomycin) on the Renal Concentration of Pb in Pb-Exposed Mice

**Figure 1:**
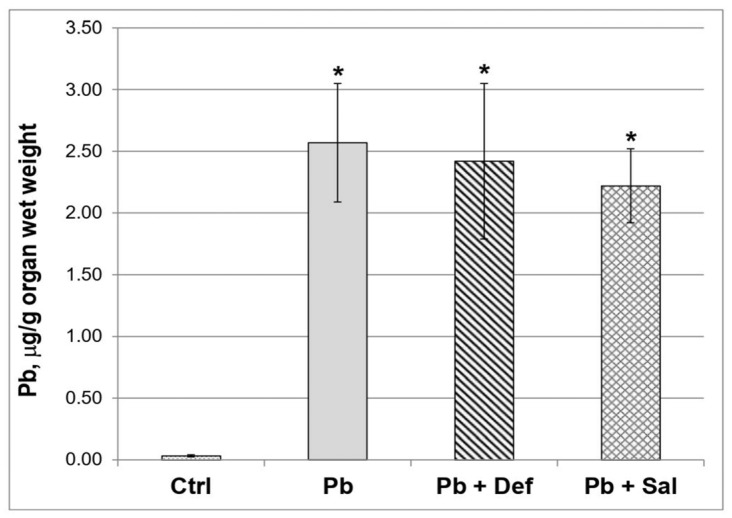
Comparative effect of deferiprone and salinomycin on the Pb content in kidneys of Pb-intoxicated mice, *n* = 6. Legend: ctrl—untreated mice; Pb—Pb-exposed group; Pb + Def—Pb-intoxicated mice, treated with deferiprone; Pb + Sal—Pb-intoxicated mice, treated with salinomycin. Asterisk denotes a significant difference compared to the untreated control group, *p* < 0.05.

The data revealed that the concentration of toxic metal in the kidneys of the mice exposed to Pb was significantly increased compared to the untreated animals (Figure 1). The administration of deferiprone to Pb-exposed mice slightly decreased the concentration of the toxic metal ion in the kidneys, as the effect reached 5.84% (*p* = 0.646) when compared to the Pb-exposed group. The effect of salinomycin administration on Pb concentration in the kidneys of Pb-exposed mice was more pronounced compared to that of deferiprone (13.62%), although it was not statistically significant compared to the Pb-exposed group (*p* = 0.162).

### 2.2. Histological Analysis

**Figure 2:**
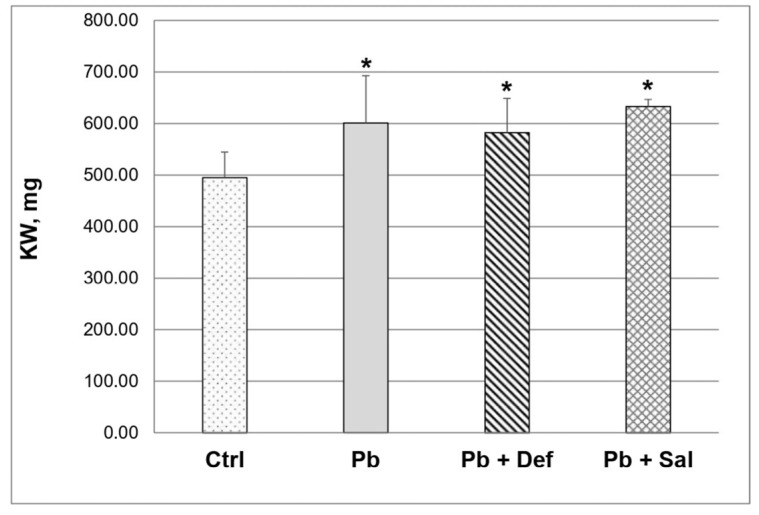
Kidney weight (KW) in control, Pb-intoxicated mice and Pb-intoxicated mice with subsequent treatment with deferiprone and/or salinomycin, *n* = 10. Asterisks denote a significant difference at *p* < 0.05 compared to the untreated mice.

Pb intoxication significantly increased (*p* < 0.05) absolute kidney weight (KW) compared to that in the untreated controls (Figure 2). Subsequent deferiprone administration reduced KW by ~3%, but it remained significantly increased compared to the untreated controls. KW continued to be significantly increased in the salinomycin-treated group as well.

**Figure 3:**
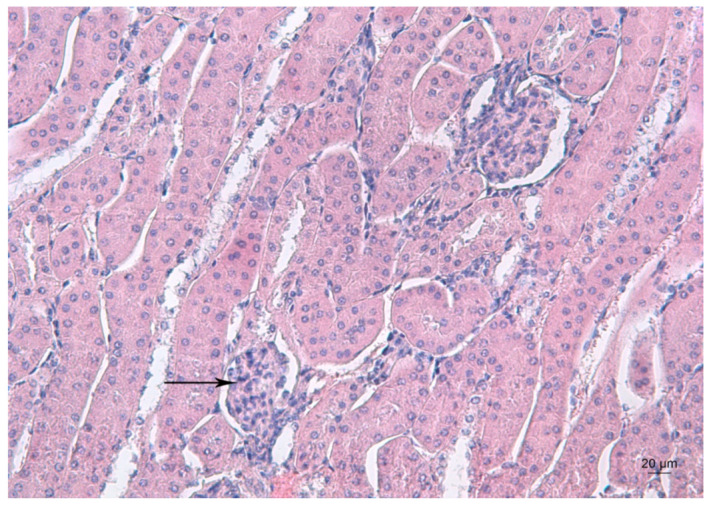
Representative photomicrograph of a kidney section of a control mouse. Arrow denotes the renal glomerulus. HE staining, ×200.

Figure 3 depicts the kidney morphology of an untreated mouse.

**Figure 4:**
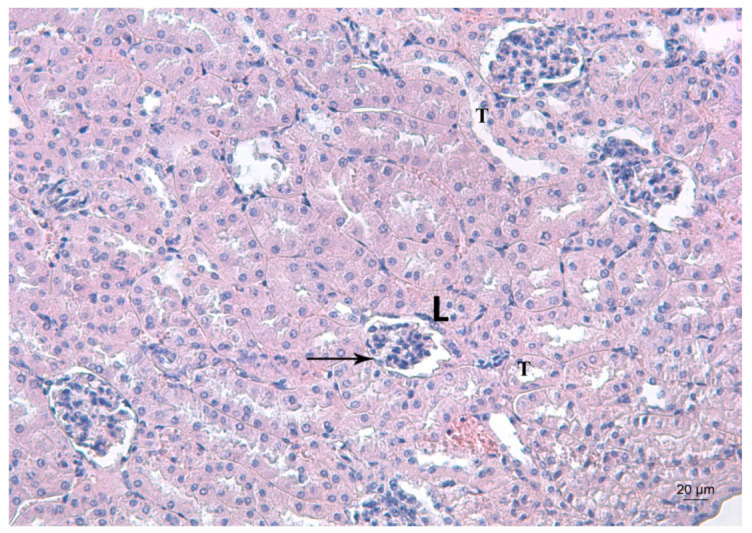
Representative photomicrograph of a kidney section of a Pb-exposed mouse. Arrow represents leukocyte infiltrations (L) near the glomerulus T—altered tubules. HE staining, ×200.

**Figure 5:**
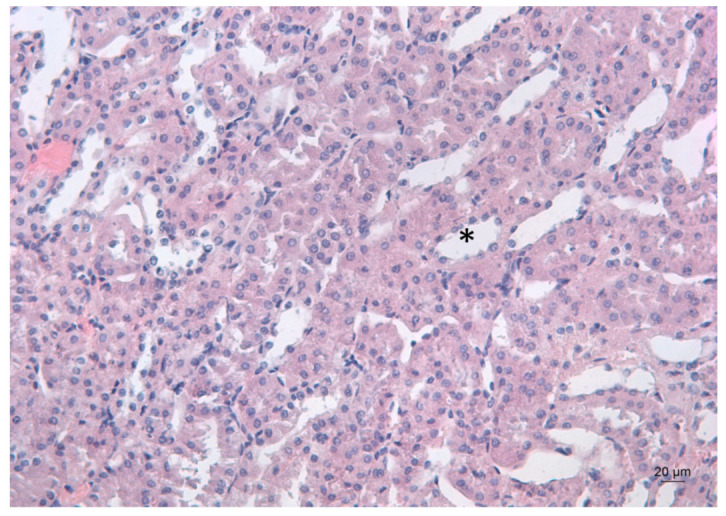
Representative photomicrograph of a kidney section of a Pb-exposed mouse, treated with deferiprone. Subsequent deferiprone administration resulted in some renal tubule dilation (*), which is often associated with degeneration. HE staining, ×200.

**Figure 6:**
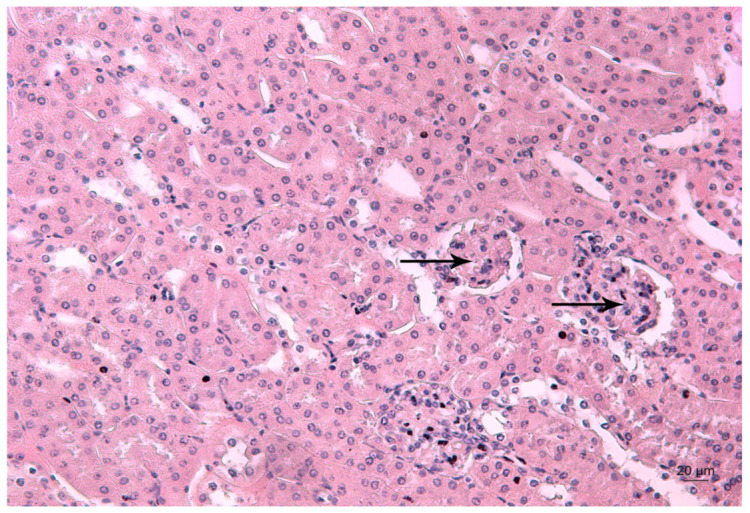
Representative photomicrograph of a kidney section of a Pb-exposed mouse, treated with salinomycin. Salinomycin administration resulted in a renal architecture close to that of the untreated controls. Arrows point to the renal glomeruli. HE staining, ×200.

Figure 4, Figure 5 and Figure 6 depict the effects of Pb, deferiprone and salinomycin on the renal tissue of Pb-exposed mice. Pb exposure of mice for 14 days induced leukocyte infiltrations near the glomerulus (Figure 4). Leukocyte infiltrations were distinguished morphologically without further analysis of the subpopulations.

Deferiprone administration to Pb-intoxicated mice resulted in the dilation of the renal tubules, which is often associated with degeneration (Figure 5).

Treatment of Pb-exposed mice with salinomycin significantly restored the renal architecture (Figure 6 compared to Figure 3).

### 2.3. Effects of Pb, Deferiprone and Salinomycin on Glomeruli Count and Glomeruli Area

The quantitative analysis of renal tissue showed that subacute Pb exposure induced a significant increase in the glomeruli count (*p* < 0.01) compared to the untreated controls (Table 1). This parameter remained significantly increased (*p* < 0.01) in Pb-exposed mice subsequently treated with deferiprone. Treatment of Pb-intoxicated mice with salinomycin significantly reduced the glomeruli count compared to the toxic controls (*p* < 0.01) and to the deferiprone-detoxified group (*p* < 0.01). In the kidneys of Pb-exposed mice subsequently treated with salinomycin, the number of glomeruli was 9.9% lower compared to the untreated controls, but the effect was not statistically significant.

The total glomeruli area (calculated as a percent of the total area of the section) significantly increased (*p* < 0.01) in Pb-intoxicated mice compared to the untreated controls (Table 1). This parameter remained significantly elevated (*p* < 0.001) in mice subsequently treated with deferiprone. The percentage of glomeruli area in Pb + Def-treated mice was significantly higher (*p* < 0.001) compared to the Pb-intoxicated mice. Subsequent treatment of Pb-exposed mice with salinomycin reduced the glomeruli area, and the parameter was lower compared to the untreated controls, but the difference was not statistically significant. The administration of salinomycin to Pb-exposed mice significantly reduced the glomeruli area compared to the toxic controls (*p* < 0.001) and to the deferiprone-detoxified group (*p* < 0.002).

The mean glomerular area (µm^2^) was significantly reduced in Pb-intoxicated mice compared to the untreated controls (*p* < 0.001) (Table 1). Treatment of Pb-exposed mice with deferiprone significantly increased the mean glomerular area compared to the toxic controls, but the average value remained lower compared to the controls. Salinomycin treatment of Pb-exposed mice significantly increased the mean glomerular area compared to the toxic controls (*p* < 0.001) and to the deferiprone-detoxified group (*p* < 0.01).

### 2.4. Effects of Pb, Deferiprone and Salinomycin on Biochemical Markers in the Sera of Experimental Mice

The exposure of mice to Pb (II) nitrate for 14 days induced a significant elevation of the serum creatinine and urea concentrations by 8.13% and 18.04%, respectively, compared to the untreated mice. Pb intoxication induced a significant decrease of 24.05% of the serum glucose concentration and an elevation of the activity of the enzyme alpha-amylase compared to the untreated mice. Treatment of Pb-exposed mice with deferiprone or salinomycin reduced the serum creatinine concentration to normal control values. However, the serum urea concentration in Pb-exposed mice subjected to treatment with deferiprone or salinomycin remained significantly higher compared to the untreated group (Table 2). The administration of deferiprone to Pb-exposed mice did not result in restoration of the serum glucose concentration to normal control values, and the average serum activity of alpha-amylase remained higher when compared to the untreated controls. Treatment of Pb-intoxicated mice with salinomycin also did not restore the serum glucose concentration to normal control values. It should be pointed out, however, that the serum activity of alpha-amylase in Pb-exposed mice subjected to treatment with salinomycin was comparable to the values in the normal control group (Table 2).

### 2.5. Effects of Pb, Deferiprone and Salinomycin on the Renal Concentrations of Essential Elements

The results for the endogenous concentrations of the essential elements are presented in Table 3. Data demonstrated that exposure of mice to Pb (II) nitrate for 14 days significantly decreased the renal concentrations of phosphorous (P) and calcium (Ca) by 12.09% and 11.07%, respectively, compared to the untreated controls. There were significantly lower values of 18.02% and 19.62%, respectively, for copper (Cu) and selenium (Se) concentrations in the kidneys of Pb-exposed mice compared to the untreated controls. The endogenous concentrations of magnesium (Mg) and iron (Fe) in the kidneys of Pb-exposed mice were not significantly affected by Pb intoxication when compared to the untreated mice.

The administration of deferiprone or salinomycin (as tetraethylammonium salt of salinomycinic acid) did not completely restore the endogenous levels of the elements P and Cu in the kidneys of Pb-exposed mice, and the values remained lower compared to the untreated controls. Treatment of Pb-exposed mice with deferiprone did not significantly decrease the concentrations of Mg and Fe in Pb-exposed mice compared to the untreated controls. Deferiprone administration restored the renal endogenous Se concentration close to normal control values. Renal Mg concentrations in Pb-exposed mice treated with salinomycin was significantly lower, by 12.79%, when compared to the values for the untreated controls. Treatment of Pb-exposed mice with salinomycin induced a significant elevation of the renal Fe concentration compared to the untreated controls, by 34.17%. The endogenous Se concentration in the kidneys of Pb-exposed mice treated with salinomycin remained significantly lower compared to the untreated controls.

The data in Table 4 demonstrate the interaction of Pb with the essential elements in the kidneys of the experimental animals. Strong positive correlations between Pb/Fe and Pb/Se were observed in the kidneys of untreated mice. Pb exposure alters the correlations between the toxic metal ion and essential elements. There were very strong negative correlations between Pb/Mg and Pb/P in the kidneys of Pb-exposed mice subjected to treatment with deferiprone. There were no significant linear correlations between the toxic metal and essential elements in the kidneys of Pb-exposed mice treated with salinomycin.

## 3. Discussion

Studies on animal models have revealed that kidneys are one of the target organs of Pb exposure [19,20,21,22]. Pb binds to low molecular proteins and penetrates the renal tubular cells by endocytosis [2,23]. Exposure to Pb has been shown to induce a significant increase in absolute kidney weight [24]. The kidney enlargement of lead-exposed rats has been associated with enhanced accumulation of lipids in the organ [24]. Our results are in a good agreement with data from the literature and demonstrated that Pb exposure of mice resulted in a significant accumulation of the toxic metal ion in the kidneys, as well as a significant increase in the absolute kidney weight when compared to the untreated controls. Pb-induced renal toxicity has been monitored by analysis of serum renal functional markers [25,26,27,28,29]. Previously published results have demonstrated that exposure of animals to Pb increased serum creatinine and urea concentrations when compared to untreated controls [6,27,28,29]. Likewise, we found significantly higher values for serum creatinine and urea concentrations in Pb-exposed mice. Reduced renal clearance and glomerular filtration rate have been proposed as possible reasons for the elevated serum creatinine and urea concentrations in Pb-exposed animals [6]. Moreover, it has been reported that serum creatinine in mice increases when the glomerular filtration rate (GFR) decreases by 70% compared to the basal values [30]. Studies have demonstrated that Pb exposure decreases serum glucose concentration [31] and alters the activity of alpha-amylase. The inhibition by lead of the glucose absorption and transport has contributed to a Pb-induced decrease in glucose concentration [31]. Conditions such as hepatitis, as well as adrenal and pituitary gland disorders, might also result in hypoglycemia [32]. Disturbed renal clearance has been proposed as a reason for elevated serum alpha-amylase activity [33]. It should be pointed out that Pb-induced elevation of the serum alpha-amylase activity could also be a result of inflammation of the pancreas [34]. Our finding on the effect of Pb exposure on serum glucose concentration and the activity of the enzyme alpha-amylase agrees well with the data from the literature [31,33] and might be associated with the Pb-induced renal alterations. Histological studies of the kidneys of Pb-intoxicated animals have shown that Pb causes both tubular and glomerular damage [6,35]. Our data corroborated the results reported in the literature and proved that Pb exposure induced pathological alterations in the renal tissue, such as tubular dilation, sloughing of the epithelium and widening of Bowman’s space of the glomeruli. The quantitative histological analysis revealed an increased glomeruli count in the region of interest in Pb-exposed mice compared to untreated mice. This may explain the increased total glomeruli area, although the mean glomerular area was reduced. The observed changes could be attributed to a compensatory response to Pb intoxication. In contrast, earlier histologic studies reported an increased glomeruli volume in response to chronic Pb intoxication [36].

In this study, we compared the efficacy of two lipophilic compounds, deferiprone and salinomycin (administered as tetraethylammonium salt of salinomycin acid) in mobilizing Pb from the kidneys of Pb-exposed mice, and we provided data about the effects of both chelating agents on Pb-induced renal alterations. We found that deferiprone administration induced a mild effect on the renal Pb concentration. Similarly, Kotyzova et al. reported that this chelating agent did not affect Pb accumulation in the kidneys of rats subjected to the administration of Pb (II) acetate and deferiprone [17]. Contrary to these results and our data, Balooch et al. [18] found that deferiprone significantly reduced renal Pb content in Pb-exposed rats by 42.34% when compared to untreated controls. In the study by Balooch et al., deferiprone was administered at an average daily dose of 300 mg/kg b.w. for 10 days, and no results about the effect of this chelating agent on Pb-induced renal damage were presented [18]. Compared to deferiprone, salinomycin was more effective in reducing the concentration of the toxic metal ion in the kidneys. It should be noted that the effect of salinomycin on Pb content in the kidneys was reproduced in independently conducted experiments. A previously published study demonstrated that the oral administration of 20 mg/kg b.w. salinomycin to mice subjected to intoxication with 80 mg/kg b.w. Pb (II) nitrate for 14 days significantly decreased the renal Pb content by 37% compared to untreated controls [15]. In the present study, the dose of the chelating agent was lower compared to that in previously published data, which explains the decreased effect of salinomycin on the renal Pb content. The data presented in this study demonstrated that neither of the chelating agents reduced the absolute kidney weight of Pb-exposed mice. The administration of deferiprone or salinomycin to Pb-exposed mice restored serum creatinine concentrations to normal control values. The serum urea and glucose concentrations in mice that were administered salinomycin remained significantly different compared to untreated controls. During the experimental protocol, we did notice reduced water consumption and body weight of the mice subjected to salinomycin treatment compared to untreated controls. Mild dehydration and loss of body weight of the mice treated with salinomycin could explain the observed effects on serum glucose and urea concentrations. It should also be pointed out that the administration of a higher dose of salinomycin (20 mg/kg b.w.) to Pb-exposed mice did not induce significant alterations of serum and urea concentrations compared to untreated controls (unpublished results). This finding supports the conclusion that the observed effects on serum urea and glucose levels in Pb-exposed mice, treated with salinomycin are not directly related to the applied chelating agent. Further, the histopathological analysis of renal tissue of Pb-exposed mice that were administered salinomycin did not reveal any significant lesions or alterations in either the tubules or glomerulus. It has been reported that salinomycin binds Pb (II) to form a complex compound of the composition [Pb(C_42_H_69_O_11_)(NO_3_)] [37]. Most likely, the ameliorative effect of salinomycin on the renal morphology of Pb-exposed mice is a result of direct chelation of the toxic metal ion by the antibiotic.

In the present study, we observed dilated renal tubules in Pb-exposed mice treated with deferiprone. This observation could be explained by direct toxic injury to the tubular epithelium. Secondary mechanisms, such as inflammation or fibrosis, might account for tubular dilation [38]. The number of the glomeruli in the ROI, as well as the total glomeruli area in kidneys of Pb-exposed mice treated with deferiprone, remained higher compared to the untreated control mice.

Recent studies have demonstrated that the essential elements could be used as biomarkers for acute renal injury [7,39]. A study by Yu et al. [7] demonstrated that exposure of mice to Pb at doses lower than 1000 mg/kg b.w. induced alterations in the homeostasis of the essential elements and concluded that the essential elements are sensitive toxicological indicators of Pb poisoning. Deficits in Mg supplementation in animals has been shown to trigger inflammation and oxidative stress [40]. Low Mg intake results in calcification of the kidneys [40]. Despite the significance of this essential element for renal function, the effect of Pb poisoning on magnesium homeostasis has been poorly studied. Pb (II) can displace Mg (II) from its binding sites in various enzymes causing an accelerated release in serum [41]. Yu et al. [7] demonstrated that the effect of Pb exposure on Mg concentration in the kidneys strongly depends on the dose of the toxic metal. An increased concentration of Mg in the kidneys has been observed in mice exposed to 1000 ppm Pb for three days [7]. Altered metabolic activity in the organs and an adaptive response to the Pb exposure are possible explanations for the enhanced Mg renal concentration [7]. Missoun et al. [31] reported that Pb-induced kidney dysfunction is associated with alterations of P and Ca homeostasis. Pb replaces Ca in Ca transporters, such as Ca channels and Ca (II)-ATPase, and inhibits its uptake. Dysregulation of Ca homeostasis is known to induce malformation of podocytes (glomerular visceral epithelial cells of the kidney) and may contribute to the development of kidney disease [42]. The toxic metal ion can also decrease the absorption of Zn (II) and Fe (II) through interaction with the divalent metal ion transporter 1 (DMT1) [41]. Yu et al. [7] demonstrated that Pb induced a dose-dependent elevation of endogenous Ca concentration and decreased the endogenous Fe concentration in the kidneys. Se is another essential element that can be affected by Pb exposure. As a component of selenoproteins and glutathione peroxidase, this element plays an important role in the antioxidant defense system [41]. A study on immature female rats revealed that Pb increased the excretion of Zn, Cu, Mn, Se and Fe in urine and in the renal cortex [9]. In our study, we found significantly lower concentrations of Ca, P, Cu and Se in the kidneys of Pb-exposed mice compared to the untreated controls. Our data are in good agreement with results from the literature [9,31] and support the conclusion that the depletion of essential elements in the kidneys of Pb-exposed animals can be considered a reason for Pb-induced renal injury. It has been demonstrated that the decreased concentration of essential elements in the renal cortex is associated with increased urinary excretion as a result of impaired tubular capacity for reabsorption and with inhibited glomerular filtration [9]. Inhibition of the absorption of the essential elements by Pb can be another explanation for the lower concentrations of Ca, Cu and Se in the blood and tissues of Pb-exposed animals [7,41].

In our study, we did not find significant correlations between Pb and the essential elements in the kidneys of Pb-exposed mice. These results demonstrated that there is no linear relationship between the Pb concentration in the kidneys of Pb-intoxicated mice and the renal endogenous concentrations of essential elements.

Treatment of Pb-exposed mice with deferiprone or salinomycin did not restore the endogenous concentrations of P and Cu in the kidneys of Pb-exposed male mice, and the values remained significantly lower compared to those of the untreated controls. In the Pb-exposed mice treated with deferiprone or salinomycin, the renal Ca concentration did not significantly differ from that of the untreated controls. The renal Se concentration in Pb-exposed mice that were treated with deferiprone was also not significantly different compared to that of the untreated mice. The ameliorative effect of deferiprone on Se concentration in the kidneys is most likely related to the restoration of the activity of the enzyme glutathione peroxidase. It was interesting to observe that deferiprone administration did not decrease the renal Fe concentration. It should be noted that the effect of deferiprone on Fe content in Pb-exposed animals has been poorly studied. Kotyzova et al., 2001 [17] and Balooch et al., 2014 [18] did not find statistically significant effects of deferiprone on the Fe content in the kidneys of Pb-exposed rats compared to the toxic control group (Pb group). Similarly, in our study, we did not observe a significant effect of deferiprone on the Fe content in Pb-exposed mice compared to the toxic group. The strong negative correlations between Pb/Mg and Pb/P in the kidneys of Pb-exposed mice treated with deferiprone demonstrated that the chelation of Pb from deferiprone in the kidneys could be significantly enhanced by mineral supplements. A slight decrease in Mg concentration in the kidneys of Pb-exposed mice treated with salinomycin was observed, which could contribute to the ability of the chelating agent to form a complex with this essential metal ion [43]. The chelating agent increased the renal concentration of iron compared to the normal control value. Most likely, salinomycin enhanced the absorption of iron.

## 4. Materials and Methods

### 4.1. Chemicals and Reagents

Biovet Ltd. (Peshtera, Bulgaria) provided the sodium salt of salinomycin (C_42_H_69_O_11_Na, CAS number: 55721-31-8, purity: >95 %) required for this study. Lead nitrate (Pb(NO_3_)_2_), tetraethylammonium hydroxide (Et_4_NOH) and diethyl ether (Et_2_O) were purchased from Merck (Darmstadt, Germany). Deferiprone (C_7_H_9_NO_2_, CAS number: 30652-11-0, purity: 98%) was purchased from Sigma Aldrich (St. Louis, MO, USA). HNO_3_ (≥69%, Rotipuran Supra, Carl Roth, Karlsruhe, Germany) and H_2_O_2_ (30%, Suprapur, Merck, Darmstadt, Germany) were used for digestion of the kidneys. All samples were diluted with ultrapure water (18.2 MΩ cm, ELGA water purification system, Purelab Ultra MK 2, UK or 18.2 MΩ cm, Milli-Q Advantage, Darmstadt, Germany). Standard solutions for calibration were purchased from LabKings (Hilversum, The Netherlands).

### 4.2. Preparation of Salinomycinic Acid

To a solution of sodium salinomycin (0.7356 g, 0.95 mmol in 20 mL Et_2_O), a solution of HCl (2 mL concentrated HCl in 20 mL H_2_O) was added. The reaction mixture was stirred for 30 min at room temperature. Both layers (organic and aqueous) were separated. The organic layer was washed with water three times. After the evaporation of the organic solvent, the compound was dissolved in acetone and isolated by precipitation with water [44]. Details about the purity and structure of the compound are given in Ivanova et al. [44].

### 4.3. Animals

The experimental protocol was performed on male 60-day-old Institute of Cancer Research (*ICR)* mice. *ICR* mice are albino mice suitable for toxicological, immunological and pharmacological studies. They have good reproductive performance, are inexpensive, robust and grow rapidly. The study was conducted at the Institute of Experimental Morphology, Pathology and Anthropology with Museum, Bulgarian Academy of Sciences according to the ARRIVE guidelines and EU Directive 2010/63/EU on animal experiments [45]. Consistent with the ARRIVE guidelines, the biochemical and ICP-MS analyses were blinded. The sample size in this study was arrived at after consultation with an expert in biostatistics and corresponds to published toxicological studies and the requirement for a precise determination of the biological effects. All animals were treated humanely. There were no visible side effects or suffering of the animals as a result of the treatments. The exposure of mice to Pb and the subsequent treatments with deferiprone or salinomycin did not cause mortality. Each animal was housed in an individual cage with a polypropylene bottom at standard conditions (light/dark cycle 12:12, constant humidity and temperature, dry bedding and quiet place with no irritation) and obtained food ad libitum. The whole experimental protocol was conducted by trained and qualified experts in animal welfare. The study was approved by the Bulgarian Agency for Food Safety, approval number: 282 from 24.09.2020. In the experimental protocol, no female mice were used because no sex-specific effects of Pb on kidney function in adults have been reported. A study by Ghorbe et al. proved that oral exposure of adult rats to Pb caused renal deficiency in both males and females [46]. The extent of the observable effects was not sex dependent. Moreover, neither of the tested chelating agents exert hormonal properties; thus, sex-dependent responses were not anticipated. It should also be pointed out that male animals have been used in most of the studies that have focused on the potential application of chelating agents as antidotes for the treatment of Pb intoxication.

### 4.4. Experimental Design

Forty male 60-day-old *ICR* mice, weighing 25–30 g, were randomly divided into four groups according to their body weight. The treatment of the animals started after one week of acclimatization. An untreated control group (Ctrl, *n* = 10) was administered distilled water for 28 days. The Pb-exposed group (Pb, *n* = 10) was treated with 80 mg/kg b.w. lead (II) nitrate for 14 days. The compound was administered per os in drinking (distilled) water. From the fifteenth day until the end of the experimental protocol, the mice received distilled water. The protocol for Pb intoxication has been previously published [15,16,47] and induced a blood lead concentration higher than 20 mg/dL [47]. The deferiprone-treated group (Pb + Def, *n* = 10) was administered an average daily dose of 20 mg/kg b.w. deferiprone for 14 days after two weeks’ exposure to Pb (II) nitrate at an average daily dose of 80 mg/kg b.w. We observed that the treatment of Pb-exposed mice with deferiprone at an average daily dose of 20 mg/kg b.w. for 14 days induced renal dilation. Therefore, we did not test higher doses of deferiprone. The salinomycin-treated group (Pb + Sal, *n* = 10) received treatment with tetraethylammonium salt of salinomycinic acid at an average daily dose of 16 mg/kg b.w. The compound was administered in drinking (distilled) water for two weeks. The administration of the chelating agent started after intoxication of mice with Pb (II) nitrate according to the procedure for the Pb-exposed group.

The solution intake was monitored daily for each animal. The dose of the compounds was calculated for each animal, every day. The average daily dose (for the duration of the treatment) for each animal was also calculated. The animals of the Pb (II)-exposed group (Pb), the Pb + salinomycin-treated group (Pb + Sal) and the Pb + deferiprone (Pb + Def)-treated group received the same doses of Pb (II) nitrate. All animals of the Pb + deferiprone-treated group received the same dose of deferiprone as well. Some variations in the solution intake were observed for salinomycin, but the differences were not significant and did not affect the experimental results.

In this protocol, no control groups treated with the chelating agents only were included. It should be noted that control groups with chelating agents or antioxidants have been included in experimental protocols to estimate the potency of the compounds to prevent damage induced by exposure to toxic metals. In post-intoxication experimental studies designed to evaluate the ameliorative effects of chelating agents or antioxidants on Pb-induced organ toxicity, such control groups have not been used [48,49]. We also have to point out that no alterations in the renal content of bio-elements in the control animals, treated with salinomycin or deferiprone only, were observed. The biochemical markers in control animals treated with salinomycin or deferiprone only also remained unchanged compared to the control samples (Supplementary Materials).

On the last day of the experimental protocol, the animals were sacrificed under light ether anesthesia, and the kidneys were excised, weighed and processed for histological and ICP-MS analyses. The kidneys of four animals from each group were processed for histological analysis, and the kidneys of six animals from each group were stored at −80 °C for ICP-MS analysis. Blood samples were collected, and sera were prepared by centrifugation at 1500 rpm for 15 min. The samples were frozen at −20 °C before analysis. The sample size used in this study corresponds to the accepted sample size in toxicological manuscripts.

### 4.5. Biochemical Analyses of Sera

The biochemical analysis of the sera from the experimental mice was conducted at the Laboratory of Toxicology, Faculty of Chemistry and Pharmacy, Sofia University “St. Kliment Ohridski” with a Mindray BA-88A biochemical analyzer. The serum biochemical markers, including creatinine (CR), urea, non-fasting glucose (Glu) and alpha-amylase, were determined according to established clinical protocols with commercially available kits (Sentinel Diagnostics, Milano, Italy).

### 4.6. ICP-MS Analysis

#### 4.6.1. Sample Preparation

Digestion was performed on the total weight of tissue samples, one kidney per tube (weight varied between 180 and 394 mg) in PFA tubes, with the addition of 6 mL 20% HNO_3_ (≥69%, Rotipuran Supra, Carl Roth, Karlsruhe, Germany) and 300 µL H_2_O_2_ (30%, Suprapur, Merck, Darmstadt, Germany). The suspensions were then placed on a hot plate and heated for six hours using a temperature program with a maximum temperature of 200 °C. After cooling, the clear solutions were transferred directly to 15 mL tubes with the remaining liquid removed from the PFA tubes by washing twice with 6 mL ultrapure water. The digested samples were diluted with Milli-Q water, resulting in nitric acid concentrations lower than 5% to result in an end volume of about 10 mL prior to ICP-MS measurement. Considering the total weight of the resulting sample solution, the dilution factor was calculated.

#### 4.6.2. Measurements

The digested samples were diluted with an ultrapure Milli-Q water (18.2 MΩ cm, ELGA water purification system, Purelab Ultra MK 2, UK or 18.2 MΩ cm, Milli-Q Advantage, Darmstadt, Germany), which resulted in nitric acid concentrations of approx. 5% prior to ICP-MS measurement. The total content of each element in the kidneys was determined with an ICP-Triple-Quadrupole MS instrument Agilent 8800 (Agilent Technologies, Tokyo, Japan), equipped with a Micro Mist nebulizer at a sample uptake rate of approximately 0.25 mL/min. Standard solutions were purchased from LabKings (Hilversum, The Netherlands). For validation of the analytical method, the certified reference material, TM-28.4 Lake Ontario water (Environment and Climate Change, Burlington, ON, Canada), was used. The monitored isotopes (24Mg, 31P, 40Ca, 56Fe, 63Cu, 64Zn, 78Se, 115In, 185Re, 208Pb) were recorded with a dwell time of 0.3 s and 10 replicates. The instrument was equipped with nickel cones and was operated at an RF power of 1550 W, with argon as the plasma gas (15 L/min), as well as the carrier gas (1.10 L/min). The Mass Hunter software package,(Workstation Software, Version B.01.03, 2016 by Agilent Technologies, Inc. (Santa Clara, CA, USA) was used for data processing.

### 4.7. Histological Analysis

Kidneys from control and treated mice were fixed in Bouin fixative for 24 h and paraffin embedded. Briefly, after fixation, the samples were dehydrated in a graded series of ethanol, cleared with xylene, impregnated in molten paraffin, embedded in fresh molten paraffin and sectioned into 5 μm thick sections using a microtome. Subsequently, the sections were stained with hematoxylin and eosin (HE) and observed on a light microscope, Leica DM 5000B (Leica Microsystems, Wetzlar, Germany).

### 4.8. Morphometric Analysis

A series of photos were taken with a research-class microscope (Leica DM5000B, Camera Leica DFC 420C and Magnification C-Mount- 0.70x- Lens 5x/0.12 NPLAN) and software Leica Application Suite Version 4.1., LASv4.1. (Leica Mircrosystems, Wetzlar, Germany). Images were saved without compression in TIFF format followed by software processing with Image Composite Editor, Version 2.0.3.0 (Microsoft Corporation, Redmont, WA, USA) to combine images into one complete image of the entire section without compression into TIFF format. Morphometric measurements were performed using Image J software, Vesrion 1.53 (Fiji modification) (NIH, Bethesda, MD, USA). Images were calibrated and checked for compliance.

Renal glomeruli were scored per sample. A region of interest (ROI) of 3000 µm^2^ in size was selected in each sample. The number of glomeruli was counted in the selected ROI.

To measure the surface area of the individual glomerulus, we used manual delineation and manual counting of each section separately, and the mean glomerular area (µm^2^) was estimated. The total glomeruli area was calculated as a percent of the total area of the section. Data were collected in Excel 2016 (Microsoft Corporation, Redmont, WA, USA) for further statistical and graphical processing.

### 4.9. Statistical Data Processing

All experimental data are represented as mean values ± standard deviation (SD). A one-way analysis of variance (ANOVA) with a Tukey’s post hoc test was conducted to determine any statistically significant difference between the groups. A Student’s *t*-test was also performed to calculate any statistically significant difference between the two groups. The differences between the groups were considered statistically significant at *p* < 0.05. The interactions between Pb and the essential elements in the kidneys were evaluated by calculating Pearson’s correlation coefficients. The statistical processing of the experimental data was performed with Excel 2016 (Microsoft corporation, Redmond, WA, USA) and the SPSS package PASW Statistics 23 (IBM corporation, Armonk, NY, USA) [50].

## 5. Conclusions

This study is the first comparative assessment of the effects of deferiprone and salinomycin on Pb-induced alterations in renal morphology. We demonstrated, for the first time, that deferiprone administration to Pb-exposed mice caused renal tubular dilation. The morphometric studies revealed an enhanced glomeruli count in the ROI, together with increased total glomeruli area in the kidneys of Pb-exposed mice treated with this chelating agent. Our results proved that salinomycin inhibited Pb-induced alterations in the renal histoarchitecture. The results for the number of glomeruli in the ROI and the total glomeruli area in Pb-intoxicated mice that were administered salinomycin also confirmed the positive effect of this chelating agent on renal function. Our study broadens the data about the effect of Pb exposure on the endogenous concentrations of essential elements in the kidneys. The study demonstrated strong negative correlations between Pb/Mg and Pb/P in the kidneys of Pb-exposed mice treated with deferiprone. For the first time, we demonstrated that salinomycin slightly decreased magnesium renal concentration. This effect should be considered when a comprehensive assessment of the efficacy of this chelating agent is needed as a potential antidote to Pb poisoning. Further studies are also needed to establish the impact of mineral supplements on the effect of deferiprone as a chelating agent for the treatment of Pb poisoning and to elucidate the effects of both chelating agents on iron homeostasis in Pb-loaded experimental models.

## Figures and Tables

**Table 1:** Glomeruli count in selected region of interest (ROI), glomeruli area as a percent of the total kidney area and mean glomeruli area (μm^2^).

Group	Glomeruli Count	Total Glomeruli Area, %	Mean Glomeruli Area, μm^2^
**Ctrl**	6.38 ± 3.02	0.42 ± 0.19	4352.61 ± 1534.34
**Pb**	12.80 ± 4.15 *	0.68 ± 0.10 *	3538.50 ± 1492.86 *
**Pb + Def**	12.67 ± 2.31 *	1.11 ± 0.22 *^,a^	3976.12 ± 1296.89 ^a^
**Pb + Sal**	5.75 ± 0.96 ^a,b^	0.38 ± 0.08 ^a,b^	4628.55 ± 1517.84 ^a,b^

*—Significant difference compared to untreated control group; ^a^—significant difference compared to toxic control group; ^b^—significant difference compared to deferiprone-detoxicated group.

**Table 2:** Comparative effect of deferiprone and salinomycin on some biochemical markers in lead-exposed mice, *n* = 10.

	CR, μmol/L	Urea, mmol/L	Glu, mmol/L	α-Amylase, U/L
**Ctrl**	28.02 ± 1.41	10.27 ± 1.57	14.22 ± 2.94	600.00 ± 64.68
**Pb**	30.50 ± 1.84 *	12.53 ± 1.93 *	10.80 ± 3.80 *	646.56 ± 55.68
**Pb + Def**	29.32 ± 2.21	12.39 ± 1.91 *	12.32 ± 2.15 *	634.71 ± 84.07
**Pb + Sal**	29.06 ± 1.79	16.42 ± 2.26 *	7.10 ± 2.11 *	608.00 ± 78.87

*—significant difference compared to the untreated control group, *p* < 0.05.

**Table 3:** Comparative effects of deferiprone and salinomycin on the concentrations of essential elements in the kidneys of Pb-exposed mice, *n* = 6.

	Mg, mg/kg	P, mg/kg	Ca, mg/kg	Fe, mg/kg	Cu, mg/kg	Se, mg/kg
**Ctrl**	244.85 ± 12.62	4373.79 ± 198.38	45.16 ± 3.45	54.05 ± 9.12	5.16 ± 0.46	1.58 ± 0.11
**Pb**	228.78 ± 13.04	3845.25 ± 197.67 *	40.16 ± 1.91 *	58.34 ± 12.60	4.23 ± 0.42 *	1.27 ± 0.14 *
**Pb + Def**	227.36 ± 13.24	3982.15 ± 163.38 *	47.59 ± 3.39	70.28 ± 7.60	4.40 ± 0.24 *	1.41 ± 0.17
**Pb + Sal**	213.54 ± 9.69 *	3794.56 ± 176.18 *	42.49 ± 2.82	82.11 ± 17.59 *	4.20 ± 0.30 *	1.31 ± 0.12 *

*—significant difference compared to the untreated control group, *p* < 0.05.

**Table 4:** Pearson’s correlation coefficients for the interaction of Pb with the essential elements in the kidneys of the experimental animals.

	Mg/Pb	P/Pb	Ca/Pb	Fe/Pb	Cu/Pb	Se/Pb
**Ctrl**	0.472	0.717	0.340	0.936 *	0.844	0.983 *
**Pb**	0.718	0.220	0.437	−0.600	0.595	0.498
**Pb + Def**	−0.866 *	−0.849 *	0.303	0.565	−0.720	−0.056
**Pb + Sal**	0.009	0.072	−0.659	0.228	0.408	0.107

*—significant difference, *p* < 0.05.

## Data Availability

All data necessary to understand or reproduce this study are included in the manuscript.

## Supplementary Materials

The following supporting information can be downloaded at: https://www.mdpi.com/article/10.3390/ijms23084368/s1.
