# Supplementary material for: Comparative Effects of Deferiprone and Salinomycin on Lead-Induced Disturbance in the Homeostasis of Intrarenal Essential Elements in Mice
Source: Int J Mol Sci. 2022 Apr 15;23(8):4368. doi: 10.3390/ijms23084368 (PMC9027580; doi:10.3390/ijms23084368)
Supplement: Supplementary file 1 [file ijms-23-04368-s001.zip › ijms-1656430-supplementary.pdf]

Table S1. Effect of deferiprone and salinomycin on the concentrations of essential elements in the kidneys of control mice*.

	**Mg, mg/kg**	**P, mg/kg**	**Ca, mg/kg**	**Fe, mg/kg**	**Cu, mg/kg**	**Zn, mg/kg**	**Se, mg/kg**
**Ctrl, N=5**	206.68±13.49	1681.70±102.21	59.58±3.50	61.64±10.33	4.80±0.67	22.24±2.21	1.24±0.11
**Ctrl+Sal*, N=3**	199.23±2.90	1669.10±99.05	59.23±2.61	57.17±9.17	4.87±0.15	20.63±0.98	1.30±0.10
**Ctrl+Def*, N=5**	197.10±10.70	1692.16±94.17	59.50±3.86	53.72±15.84	5.38±1.16	22.26±1.41	1.16±0.17

* control mice obtained salinomycin (average daily dose 16 mg/kg BW) or deferiprone (average daily dose 150 mg/kg BW) for 14 days. The treatment was conducted from the 15th to 28th day of the experimental protocol. Both compounds were administered orally. Administration of salinomycin or deferiprone to untreated control mice did not induce significant alterations of intrarenal essential elements' content compared to the control values.

Table S2. Effect of deferiprone and salinomycin on some biochemical markers in control mice*.

	**CR, μmol/L**	**Urea, μmol/L**	**Glucose, mmol/L**
**Ctrl, N=10**	24.11±1.31	10.66±2.99	11.06±3.11
**Ctrl+Sal*, N=7**	22.60±2.63	11.07±1.23	11.30±1.44
**Ctrl+Def*, N=10**	24.30±2.10	12.32±2.08	8.08±3.39

* control mice obtained salinomycin (average daily dose 16 mg/kg BW) or deferiprone (average daily dose 150 mg/kg BW) for 14 days. The treatment was conducted from the 15th to 28th day of the experimental protocol. Administration of salinomycin or deferiprone to untreated control mice did not induce significant alterations of the serum concentrations of creatinine, urea and glucose.
